# Intravesical Bacillus Calmette-Guérin (BCG) Immunotherapy-Related Pneumonitis: A Case Report

**DOI:** 10.7759/cureus.91171

**Published:** 2025-08-28

**Authors:** Nevra Gullu Arslan, Sengul Aksakal

**Affiliations:** 1 Department of Pulmonary Medicine, Samsun Education and Research Hospital, Samsun, TUR; 2 Clinic of Alergy and Immunology, Samsun Education and Research Hospital, Samsun, TUR

**Keywords:** bronchoalveolar lavage, cd4 t cells, complication, intravesical bcg, pneumonitis

## Abstract

Intravesical bacillus Calmette-Guérin (BCG) immunotherapy is widely used as a standard adjuvant treatment for non-muscle invasive bladder cancer. The exact mechanism of BCG action and the mechanisms responsible for early and late complications after intravesical BCG treatment remain poorly understood, and it is still unclear in which patients local or systemic adverse effects will develop. The patient who received intravesical BCG treatment for six weeks presented with complaints of arthralgia, loss of appetite, fatigue, and intermittent low-grade fever. High-resolution chest computed tomography showed bilateral ground-glass opacities and showed no response to antiviral or antimicrobial treatments. Bronchoalveolar lavage flow cytometry demonstrated a markedly elevated CD4/CD8 ratio (6:1). Based on clinical, radiologic, and immunologic findings, pneumonitis secondary to BCG-related inflammation (BCG-itis) was diagnosed. Differentiating infectious from immune-mediated adverse events in BCG-itis can be challenging.

## Introduction

Bacillus Calmette-Guérin (BCG), a live attenuated strain of *Mycobacterium bovis*, was originally developed as a tuberculosis vaccine and later adopted as adjuvant therapy for superficial bladder cancer [1]. Intravesical BCG following transurethral resection of bladder tumor (TURBT) is the standard treatment for intermediate- and high-risk non-muscle-invasive bladder cancer and carcinoma in situ, reducing recurrence and progression rates [2]. Although its exact mechanism is unclear, BCG stimulates a strong cytotoxic T-cell response and granulomatous inflammation [3], while B cells may enhance the innate immune response and promote tumor cell apoptosis by upregulating programmed death-ligand 1 (PD-L1) expression [4]. The exaggerated T-cell response may lead to BCG-related inflammation (BCG-itis), resulting in local or systemic complications in either the early or late period.

Local complications, such as cystitis, urinary frequency, hematuria, incontinence, spinal tuberculosis, or systemic effects such as malaise, fever, hepatitis, purpura, and septicemia, occur in about 8% of cases. Pneumonitis after intravesical BCG affects fewer than 1% of patients [5]. These complications may arise concurrently or months after BCG instillation and are believed to result from active infection, hypersensitivity, or granuloma formation resembling tuberculosis [6]. Early symptoms, such as skin rash and arthralgia, are usually self-limited and last only a few days. Severe complications are rare and can occur during or several years after treatment [7].

Diagnosing BCG-related complications is challenging, as culture or histological confirmation is often lacking. Therefore, a history of BCG therapy is essential to avoid delays in diagnosis. Understanding BCG’s immunological effects may clarify the mechanisms behind its side effects. Based on the findings recorded in this case, we aimed to evaluate the immune mechanisms that may cause BCG-associated pneumonitis.

## Case presentation

**Figure 1:**
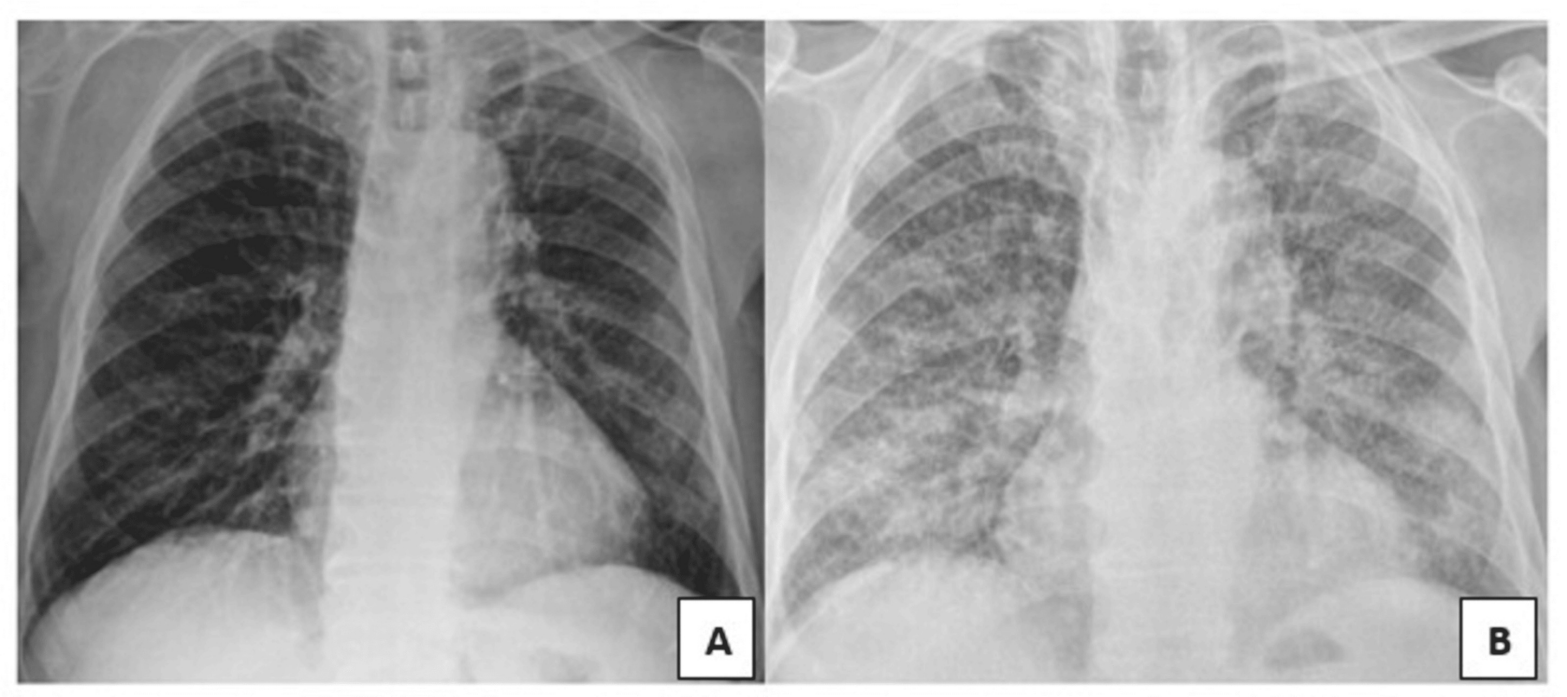
Chest X-ray. (A) Normal posteroanterior chest radiograph in first application. (B) Bilateral basal heterogeneous infiltrates

An 80-year-old male patient presented to an external infectious disease clinic with complaints of fatigue, loss of appetite, intermittent vomiting, and diffuse joint pain lasting for two weeks. He had a known history of hypertension and had been receiving intravesical BCG therapy for bladder cancer for the past six weeks. Physical examination was unremarkable. Chest X-ray was unremarkable (Figure 1A).

In the patient’s initial hospitalization, during laboratory tests, creatinine, C-reactive protein, sedimentation, and gamma-glutamyl transferase (GGT) values were elevated (Table 1). Tests for viral hepatitis were negative as well.

**Table 1:** Laboratory findings. ALT: alanine transaminase; AST: aspartate aminotransferase; CRP: C-reactive protein; GGT: gamma-glutamyl transferase; WBC: white blood cell

Laboratory value	Normal range	Initial	Follow-up
Creatinine	0.7-1.2 mg/dL	1.3	0.9
WBC	4-11 x 10 ^3^/mm^3^	8.69	7.8
AST	0-33 U/L	30	31
ALT	0-45 U/L	32	71
GGT	5-36 U/L	231	110
CRP	0-0.5 mg/dL	8.5	2.6
Sedimentation	0-15/20 mm/h	59	48

Due to intermittent fever, the patient was admitted for infectious disease evaluation. Empiric antiviral and quinolone treatments were started despite negative cultures. Persistent low-grade fever and unchanged acute phase reactants prompted a chest X-ray showing bilateral diffuse reticular infiltrates (Figure 1B). Antibiotics were escalated to piperacillin-tazobactam 4.5 g three times a day for five days, and then the patient was referred to our clinic.

**Figure 2:**
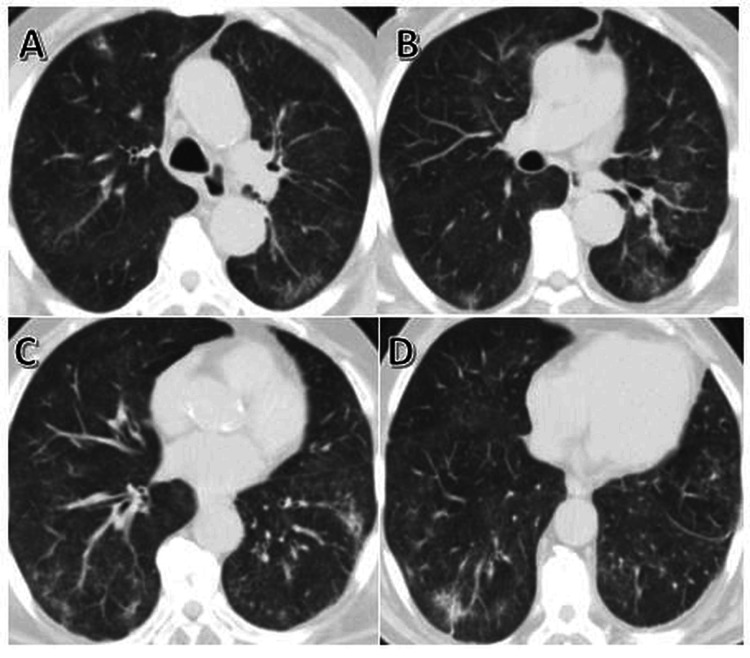
Thorax computed tomography. (A-D) Bilateral patchy ground-glass infiltrates

At admission, the patient still had fatigue and arthralgia; vital signs were stable, with rare crackles in the lung bases. High-resolution chest CT showed bilateral patchy ground-glass opacities (Figure 2). In laboratory tests, C-reactive protein, sedimentation, and alanine transaminase (ALT) were elevated (Table 1). Bronchoscopy was planned.

Bronchoalveolar lavage (BAL) was performed in the right middle lobe. Microbiological studies, including bacterial cultures, acid-fast bacilli (AFB) smear and culture, tuberculosis polymerase chain reaction (PCR), and viral PCR panel of respiratory pathogens (coronavirus and subtypes, influenza A-B, parainfluenza and subtypes, *Metapneumovirus*, rhinovirus, *Enterovirus*, respiratory syncytial virus (RSV)), were all negative. Cytological evaluation and cell count of the BAL fluid revealed abundant macrophages with scattered multinucleated giant cells, lymphocytes, and mixed inflammatory cells, with no AFB. Flow cytometry showed that 50% of cells were lymphocytes, with a CD4+ T-cell predominance (CD4: 83%, CD8: 14%, CD4/CD8 ratio: 6).

Based on clinical, radiologic, and pathological findings, BCG-related lymphocytic pneumonitis was suspected. The patient's piperasilin-tazobactam treatment, which was initiated at an external center, was continued and completed to a total duration of 14 days. The patient was started on methylprednisolone at a dose of 1 mg/kg/day for five days, followed by maintenance therapy at 0.5 mg/kg/day without concurrent antibiotic therapy. A rapid clinical response was observed under steroid treatment, and the patient was discharged on an oral corticosteroid regimen. At the one-month follow-up, near-complete radiological regression was achieved. The dose was tapered off and discontinued within three months.

## Discussion

Intravesical BCG administration and its mechanism of action used as adjuvant immunotherapy in bladder tumors have received significant attention due to BCG intolerance, BCG resistance, BCG relapse, and other serious side effects. As in our case, an immediate systemic reaction following BCG instillation, inability to isolate the causative agent microbiologically or cytopathologically, and a rapid response to steroid therapy should raise suspicion for BCG-itis.

The diagnosis and management of BCG-related complications remain challenging. The variability in the onset of symptoms after the last instillation, together with radiological and clinical manifestations that may mimic malignancy or other infections, has emphasized the need for individualized, case-based management. Treatment decisions are guided by the severity, extent, and localization (local or systemic) of the complication, as well as the invasiveness of the biopsy procedure. In cases with a strong suspicion of severe systemic reactivated infection unresponsive to conventional antibacterial therapy - even in the absence of definitive diagnostic evidence - antimycobacterial therapy is recommended; in severe cases progressing to sepsis, a combination of antimycobacterial agents with corticosteroids is advised [8].

In local complications with non-diagnostic urine culture results and no response to antibiotics, empirical treatment targeting BCG infection may be considered. If there is no clinical improvement with this approach, biopsy may be performed; while granulomatous reaction per se does not require treatment, management strategies may vary depending on the affected organ (e.g., a testicular abscess unresponsive to antimycobacterial therapy may ultimately require orchiectomy) [8].

In a case series by Gonzalez et al. [9], late-onset intravesical BCG complications with localized symptoms were microbiologically confirmed in about two-thirds of cases, while only one-third of early systemic cases had such confirmation. This suggests systemic reactions result from acute inflammation to a low-virulence pathogen, causing a granulomatous response, whereas localized disease stems from chronic infection or latent reactivation [6]. Early and late immune responses to the infectious agent may also contribute to BCG-related complications.

Beyond its direct cytotoxic effects, BCG induces apoptosis, necrocytosis, and oxidative stress in tumor cells. Acting as a pathogen-associated molecular pattern (PAMP), BCG activates pattern recognition receptors (PRRs) such as Toll-like receptors (TLRs) on bladder tumor cells, macrophages, and antigen-presenting cells (APCs). The myeloid differentiation primary response 88 (MyD88) pathway is a key signaling route in the innate immune system, particularly involved in TLR and IL-1 receptor (IL-1R) signaling. Activation of TLRs leads to nuclear translocation of NF-κB and transcription of pro-inflammatory cytokines that sustain rapid inflammatory responses against pathogens, playing a critical role in early defense [10]. Genetically modified bacterial therapies have gained attention recently, but BCG remains the only bacterial drug approved and widely used in cancer treatment [11].

CD4+ T-helper cells support immune defense by activating B cells and macrophages and enhancing CD8+ T-cell activity. Predominance of CD4+ T lymphocytes in bronchoalveolar lavage fluid indicates a helper T-cell-driven immune response, as seen in granulomatous diseases, such as sarcoidosis, autoimmune and opportunistic infections, or in chronic stages of certain interstitial lung diseases. Israel-Biet et al. [12] reported three pulmonary BCG complication cases showing alveolar lymphocytosis and elevated CD4/CD8 ratios in BAL, with no mycobacteria detected - suggesting an exaggerated immune response rather than active infection. Similarly, in our case, no pathogenic organism was isolated, and we believe that the elevated CD4/CD8 ratio reflects an excessive inflammatory response that has developed to control a potential tuberculosis infection capable of eliciting a granulomatous reaction.

Additionally, as a result of BCG internalization or macrophage-mediated phagocytosis, APCs process BCG and present its antigens to CD4+ and CD8+T cells by binding them to major histocompatibility complex class II (MHC-II) molecules. This mechanism promotes the activation of adaptive immune responses. Following antigen presentation by APCs, CD4+ T cells release a range of cytokines, including IL-1, IL-2, IL-6, IL-8, IL-10, IL-12, IL-17, IL-18, TNF-α, IFN-γ, and GM-CSF. Tumor cells and innate immune cells also produce large amounts of cytokines upon stimulation by BCG. Notably, many of these cytokines are Th1-type, creating a cytokine milieu that is particularly favorable for the activation of CD8+ T cells [13,14].

There are no clear recommendations to treat intravesical BCG-related adverse events. Treatment usually varies depending on the extent (local versus systemic) and severity of symptoms; antipyretics, nonsteroidal anti-inflammatory drugs to antituberculosis therapy and systemic corticosteroids can be used.

## Conclusions

This case underscores the diagnostic challenges associated with BCG-related adverse effects, which are believed to arise from immune dysregulation and may present as active infection, hypersensitivity reactions, or granuloma formation. The timing of symptom onset is critical, as it often reflects the underlying pathophysiological mechanism. Due to the lack of specific clinical features and the frequent absence of definitive microbiological or histopathological findings, establishing a diagnosis can be difficult. In this patient, the BAL findings point to an early systemic reaction following intravesical BCG therapy, supporting the hypothesis of a hypersensitivity response triggered by the immune system’s attempt to control systemic dissemination.
